# Pharmacological Programming of Endosomal Signaling Activated by Small Molecule Ligands of the Follicle Stimulating Hormone Receptor

**DOI:** 10.3389/fphar.2020.593492

**Published:** 2020-11-30

**Authors:** Silvia Sposini, Francesco De Pascali, Rachel Richardson, Niamh S. Sayers, David Perrais, Henry N. Yu, Stephen Palmer, Selva Nataraja, Eric Reiter, Aylin C. Hanyaloglu

**Affiliations:** ^1^ Institute of Reproductive and Developmental Biology, Department of Metabolism, Digestion and Reproduction, Imperial College London, London, United Kingdom; ^2^ University of Bordeaux, CNRS, Interdisciplinary Institute for Neuroscience, IINS, UMR 5297, Bordeaux, France; ^3^ Physiologie de la Reproduction et des Comportements (PRC), Institut National de Recherche pour l’Agriculture, l’Alimentation et l’Environnement (INRAE), Centre National de la Recherche Scientifique (CNRS), Université de Tours, Institut Français du Cheval et de l'Equitation (IFCE), Nouzilly, France; ^4^ CanWell Pharma Inc., Wellesley, MA, United States; ^5^ Department of Pathology and Immunology, Baylor College of Medicine, Houston, TX, United States; ^6^ Mitobridge Inc., Cambridge, MA, United States

**Keywords:** G protein coupled receptor, endosome, follicle-stimulating hormone receptor, APPL1, allosteric ligand

## Abstract

Follicle-stimulating hormone receptor (FSHR) is a G protein-coupled receptor (GPCR) with pivotal roles in reproduction. One key mechanism dictating the signal activity of GPCRs is membrane trafficking. After binding its hormone FSH, FSHR undergoes internalization to very early endosomes (VEEs) for its acute signaling and sorting to a rapid recycling pathway. The VEE is a heterogeneous compartment containing the Adaptor Protein Phosphotyrosine Interacting with Pleckstrin homology Domain and Leucine Zipper 1 (APPL1) with distinct functions in regulating endosomal Gαs/cAMP signaling and rapid recycling. Low molecular weight (LMW) allosteric FSHR ligands were developed for use in assisted reproductive technology yet could also provide novel pharmacological tools to study FSHR. Given the critical nature of receptor internalization and endosomal signaling for FSHR activity, we assessed whether these compounds exhibit differential abilities to alter receptor endosomal trafficking and signaling within the VEE. Two chemically distinct LMW agonists (benzamide, termed B3 and thiazolidinone, termed T1) were employed. T1 was able to induce a greater level of cAMP than FSH and B3. As cAMP signaling drives gonadotrophin hormone receptor recycling, rapid exocytic events were evaluated at single event resolution. Strikingly, T1 was able to induce a 3-fold increase in recycling events compared to FSH and two-fold more compared to B3. As T1-induced internalization was only marginally greater, the dramatic increase in recycling and cAMP signaling may be due to additional mechanisms. All compounds exhibited a similar requirement for receptor internalization to increase cAMP and proportion of FSHR endosomes with active Gαs, suggesting regulation of cAMP signaling induced by T1 may be altered. APPL1 plays a central role for GPCRs targeted to the VEE, and indeed, loss of APPL1 inhibited FSH-induced recycling and increased endosomal cAMP signaling. While T1-induced FSHR recycling was APPL1-dependent, its elevated cAMP signaling was only partially increased following APPL1 knockdown. Unexpectedly, B3 altered the dependence of FSHR to APPL1 in an opposing manner, whereby its endosomal signaling was negatively regulated by APPL1, while B3-induced FSHR recycling was APPL1-independent. Overall, FSHR allosteric compounds have the potential to re-program FSHR activity via altering engagement with VEE machinery and also suggests that these two distinct functions of APPL1 can potentially be selected pharmacologically.

## Introduction

Membrane trafficking of G protein-coupled receptors (GPCRs) tightly orchestrates the signaling from this superfamily of signaling receptors. In turn, receptor signaling is known to drive or regulate GPCR trafficking. This inter-relationship is further highlighted through the ability of a growing number of GPCRs to not only activate heterotrimeric G protein signaling from the plasma membrane, but also internal membrane compartments including the endosomal membrane (Thomsen et al., 2018; Caengprasath and Hanyaloglu, 2019; Weinberg et al., 2019; Sutkeviciute and Vilardaga, 2020). Thus, the spatial control of GPCR/G protein signaling represents a fundamental mechanism for how cells can both diversify signaling and decode distinct functions from common upstream second messenger signaling pathways. However, our understanding of the impact of that spatially directed G protein signaling has on downstream physiological and pathophysiological functions is limited, yet, could provide novel therapeutic strategies in targeting these receptors.

Endosomal signaling of GPCRs can alter the temporal profile of G protein signaling, such that the ligand-induced internalization of receptors to endosomes generates a “second wave” or persistent second messenger signaling profile. This has been demonstrated for distinct Gαs-coupled GPCRs such as thyrotropin-stimulated hormone receptor, parathyroid-stimulating hormone receptor and the β2-adrenergic receptor (Calebiro et al., 2009; Ferrandon et al., 2009; Irannejad et al., 2013), but also GPCRs coupled to other G protein pathways such as the kisspeptin receptor and sphingosine-1 phosphate receptor (Mullershausen et al., 2009; Min et al., 2014). In contrast, we and others have demonstrated that ligand-induced endocytosis can also be required for acute G protein signaling of certain GPCRs (Kotowski et al., 2011; Jean-Alphonse et al., 2014; Sposini et al., 2017; Caengprasath et al., 2020). We have identified that many of these receptors are sorted to an endosomal compartment termed the Very Early Endosome (VEE). The VEE is distinct from the early endosome (EE), which classically represents the primary sorting station for various membrane cargo. VEEs are physically smaller than EEs (∼a third of the size), are closer to the plasma membrane and lack the classic EE markers such as early endosomal autoantigen 1 (EEA1), Rab5 and phosphatidylinositol 3-phosphate (Jean-Alphonse et al., 2014). Additionally, a subpopulation of VEEs colocalize with the adaptor protein APPL1 (Adaptor protein, Phosphotyrosine Interacting with Pleckstrin homology Domain and Leucine Zipper 1), which has two distinct functions for GPCRs that traffic to this compartment; driving rapid receptor recycling from the VEE to the plasma membrane, and negative regulation of endosomal G protein signaling (Sposini et al., 2017). This regulation by APPL1 is a specific feature of VEE-localized GPCRs. The luteinizing hormone receptor (LHR) represents our “prototype” for VEE-localized GPCRs, however, the β1-adrenergic receptor, free fatty acid receptor 2 and follicle-stimulating hormone receptor (FSHR), are all sorted to and regulated by the VEE (Jean-Alphonse et al., 2014; Sposini et al., 2017; Caengprasath et al., 2020).

FSHR plays pivotal roles in the regulation of male and female reproduction. Although a Class A/rhodopsin GPCR, FSHR has a very large extracellular, horseshoe-shaped N-terminus through which it binds their orthosteric ligands (Fan and Hendrickson, 2005; Jiang et al., 2014). This determines conformational changes first in the receptor’s core then to its cognate G protein to initiate intracellular signaling cascades, the classic pathway of which, is activation of Gαs followed by increases in intracellular cAMP and subsequent PKA activation (Casarini and Crepieux, 2019). The glycoprotein hormone FSH is secreted by the gonadotropes of the anterior pituitary gland and activates FSHR expressed in the Sertoli cells of the testes and granulosa cells of the ovarian follicle. FSH-mediated activation of FSHR regulates spermatogenesis in males and stimulation of follicular growth and estrogen production in females (Kaprara and Huhtaniemi, 2018). For its key roles in female reproduction, FSHR is the target for infertility treatment and in assisted reproductive technology (ART) using recombinant FSH. However, low molecular weight (LMW) allosteric compounds targeting gonadotropin receptors have been developed for their potential to increase patient convenience in ART as these molecules would be orally available, offer a synergistic treatment with FSH for patients that respond poorly to recombinant FSH and reduce the risk of ovarian hyperstimulation syndrome, observed with high doses of recombinant gonadotropins (Nataraja et al., 2015; Anderson et al., 2018). From a cell biological perspective, the development of such compounds also offers advantageous tools to further understand key mechanisms that shape the signaling of these receptors, such as receptor trafficking and endosomal signaling.

Among glycoprotein receptors, most of the LMW allosteric compounds developed so far target FSHR; these include positive allosteric modulators (Sriraman et al., 2014; Yu et al., 2014), negative allosteric modulators (Dias et al., 2011; Dias et al., 2014) and allosteric agonists and antagonists (Yanofsky et al., 2006; van Koppen et al., 2013). While pre-clinical studies in animal models for these LMW agonists showed promise, the only molecule that entered clinical development failed to demonstrate follicular development in healthy patients (Gerrits et al., 2016). Given the recent advances in our understanding of GPCR signaling, including the requirement of these receptors to signal from distinct intracellular compartments, how such compounds engage mechanisms such as trafficking and endosomal signaling remains to be determined yet could offer a novel developmental avenue for the future application of such compounds.

In this study, we took advantage of LMW FSHR ligands from two distinct chemical classes, to assess whether these compounds could alter receptor endosomal signaling via impacting its trafficking compared to FSH. We demonstrate that LMW agonistic compounds exhibit enhanced endosomal signaling and recycling due to a differential sensitivity to APPL1 regulation. Such ligands offer novel potent tools to pharmacologically program FSHR to specific sorting and/or signaling outputs by their differential ability to engage the VEE machinery.

## Materials and Methods

### Reagents

**FIGURE 1:**
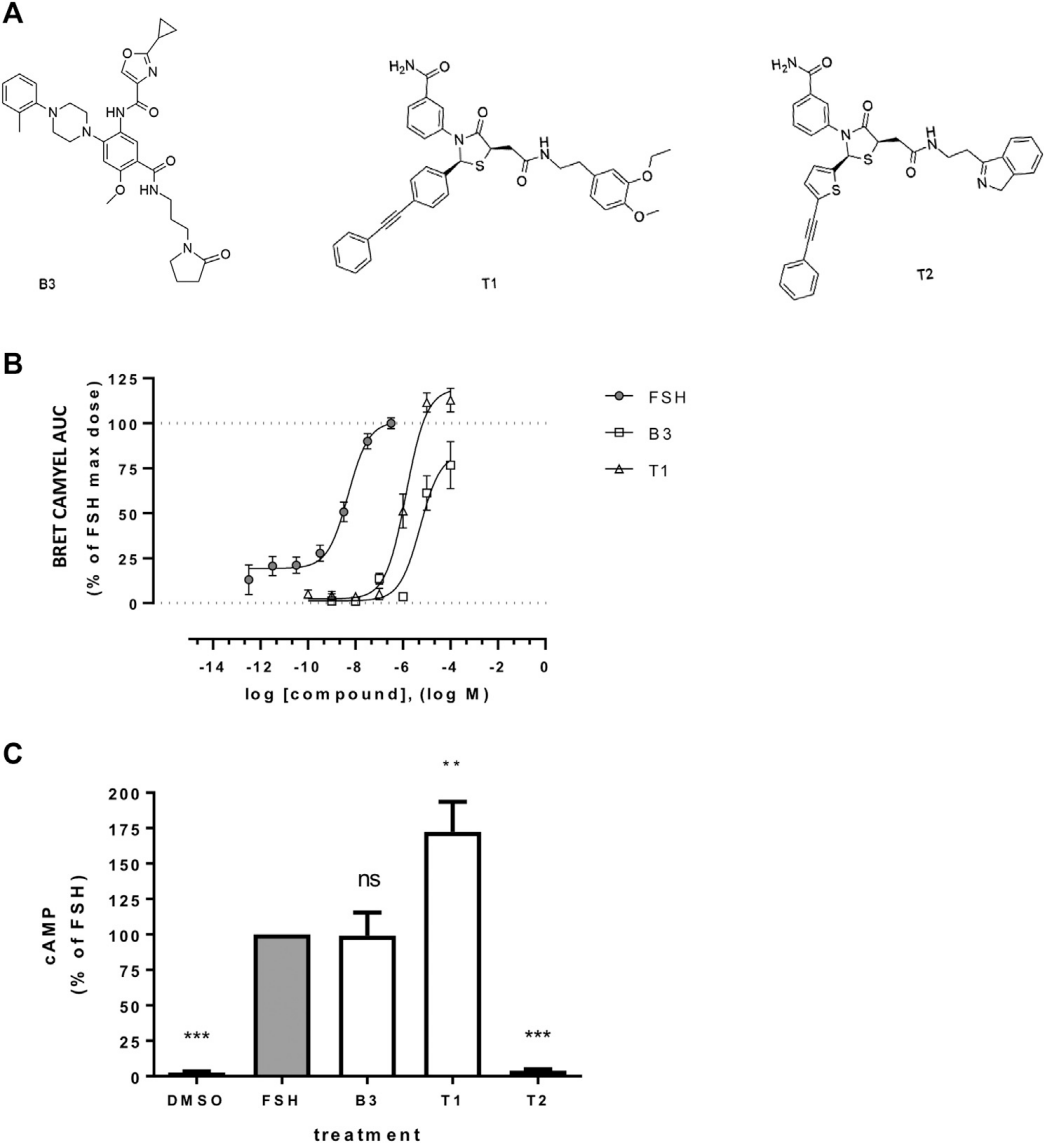
Effect of LMW allosteric compounds of FSHR to activate cAMP signaling **(A)** Chemical structures of compounds used in this study: the benzamide B3 and the thiazolidinone derivates T1 and T2. **(B)** Intracellular cAMP levels measured in cells transiently transfected with the cAMP BRET sensor CAMYEL and FSHR stimulated with increasing concentrations of FSH, B3 or T1. For each concentration considered, the area under the curve (AUC) was extrapolated from the signal recorded following 20 min stimulation and plotted as concentration/activity curves. Concentrations of FSH were −12.5 to −6.5 log M while B3 and T1 were −10 to −4 log M, at half-log dilutions. Data were normalized considering FSH maximal response as 100%. n = 6 independent experiments. **(C)** Intracellular levels of cAMP measured via HTRF in cells expressing FLAG-FSHR and stimulation with either DMSO, FSH (10 nM), B3, T1 or T2 (10 μM) for 5 min. n = 4 independent experiments; One-way ANOVA: **p < 0.01, ***p < 0.001.

The FSHR low molecular weight ligands (B3, T1, T2) were provided by TocopheRx (Burlington, MA, United States). Purity of compounds ranged from 95–97%. Chemical structures are shown in Figure 1A. Ligands were diluted in sterile dimethyl sulfoxide (DMSO, Sigma Aldrich) for conservation at +4°C then diluted in DMEM or imaging medium at appropriate concentrations for stimulations. Human pituitary FSH (A.F. Parlow, National Hormone and Peptide Program, Harbor-UCLA Medical Center, purity ≥95%) was diluted in PBS and stored at −20°C then diluted in DMEM or imaging medium at appropriate concentrations for stimulations. The antibodies used were: mouse anti-FLAG (M1, Sigma); rabbit anti-APPL1 (Cell Signaling Technology); rabbit anti-EEA1 (Cell Signaling Technology); mouse anti-GAPDH (Millipore); goat anti-rabbit and anti-mouse AlexaFluor 488, 555, 568, and 647 (Thermo Fisher); and goat anti-rabbit and anti-mouse horseradish peroxidase (HRP, Thermo Fisher Scientific). Dyngo-4a (Abcam) was diluted in DMSO and stored at −20°C protected from light, then used at 30 μM (30 min pre-treatment) in DMEM. Coelenterazine H (Interchim, purity >98%) was diluted in methanol and stored at −20°C protected from light.

FLAG-hFSHR has been previously described (Jean-Alphonse et al., 2014). Nb37-GFP and SEP-hB2AR were kind gifts from Mark von Zastrow (University of California, San Francisco, United States). SEP-FSHR was obtained as follows: SEP was subcloned from SEP-B2AR using AgeI and ligated into FLAG-hFSHR, containing an AgeI restriction site in the FLAG sequence created by site-directed mutagenesis (QuikChange, Stratagene) using oligos corresponding to GTG​TGG​TCT​CCG​ATT​ACA​CCG​GTG​ATG​ATG​ATA​AGC​GAG​C. FSHR C-terminally fused with donor Rluc8 (FSHR-Rluc8) was previously described (Ayoub et al., 2015). The BRET-based cAMP sensor CAMYEL was kindly provided by L.I. Jiang (University of Texas, Texas, United States) (Jiang et al., 2007).

siRNA-mediated knockdown of APPL1 was achieved by transfection of duplex RNA oligos (Life Technologies) corresponding to GAC​AAG​GTC​TTT​ACT​AGG​TGT​ATT​T. Control cells were transfected with non-sense duplex RNA oligos (AAT​TCT​CCG​AAC​GTG​TCA​CG).

### Cell Culture and Transfection

HEK 293 cells (ATCC) were maintained in DMEM containing 10% FBS and penicillin/streptomycin (100 U/mL) at 37°C in 5% CO_2_. Both transient and stable transfections of DNA were performed with Lipofectamine 2000 (Life Technologies). Transfection of siRNA was performed using RNAiMAX (Life Technologies). For transient expression, cells were assayed 48- or 96-h post-DNA and siRNA transfection, respectively.

### Confocal Imaging

Receptor imaging in live or fixed cells was monitored by treating cells with mouse anti-FLAG antibody (15 min, 37°C) in phenol-red-free DMEM prior to agonist treatment. Cells were washed three times in PBS/0.04% EDTA to remove FLAG antibody bound to the remaining surface receptors and then fixed (4% paraformaldehyde/PBS). Cells were permeabilized and treated with goat anti-mouse secondary antibodies conjugated to Alexa Fluor 488 or 555 and imaged using a TCS-SP5 confocal microscope (Leica) with a 63 × 1.4 numerical aperture (NA) objective. Leica LAS AF image acquisition software was utilized. All subsequent raw-image files were analyzed using ImageJ or LAS AF Lite (Leica) to measure levels of co-localization. Briefly, co-localization was assessed by a manual object-based analysis where first FSHR endosomes (objects) were identified and defined as ROIs, then presence of either APPL1, EEA1 or Nb37 was tested in those ROIs. For 3-colour TIRF this was confirmed by line-scan analysis.

### cAMP Signaling Assays

Measurement of whole-cell cAMP was carried out with the HTRF-based cAMP Dynamic 2 kit (Cisbio Bioassays) as per manufacturer’s instructions. Cells were ligand treated in triplicate, and experiments were repeated at least three times. All cAMP concentrations were corrected for protein levels.

In order to measure cAMP real-time response in living cells, HEK293 cells were transiently transfected with two plasmids coding for FSHR and the BRET-based cAMP sensor CAMYEL (Jiang et al., 2007). Forty hours after transfection, BRET measurements were immediately performed upon addition of increasing concentrations of FSH (−12.5 to −6.5 log M) or of LMW ligands (−10 to −4 log M) and 5 μM of coelenterazine H. Signals were recorded for 20 min in a Mithras LB plate reader (Berthold Technologies GmbH & Co. Wildbad, Germany). Concentration/activity curves were generated by extrapolating the area under the curve (AUC) for each concentration considered over the time course, to which was subtracted the AUC of the unstimulated basal condition. Normalization was performed considering the signal of FSH maximal concentration as 100%.

### Total Internal Reflection-Fluorescence Microscopy

Cells were imaged using an Elyra PS.1 AxioObserver Z1 motorized inverted microscope with a scientific complementary metal-oxide-semiconductor (sCMOS) or electron-multiplying charge-coupled device (EMCCD) camera and an alpha Plan-Apochromat 100×/1.46 Oil DIC M27 Elyra objective (Zeiss), with solid-state lasers of 488, 561, and/or 642 nm as light sources. For live-cell imaging, cells were imaged for 1 min at a frame rate of 10 frames per second (fps) at 37°C in phenol-red-free Opti-MEM supplemented with HEPES (Life Technologies). ZEN Lite image acquisition software was utilized to collect time-lapse movies and analyzed as tiff stacks using the ImageJ plugin Time Series Analyzer. The number of exocytic events were counted manually by visual inspection and was normalized by cell area. Maximum intensity projections, kymographs and fluorescence intensities were generated using ImageJ. For fixed-cell imaging, cells were prepared as described for confocal imaging. Counting of FSHR-, APPL1-, EEA1-, Nb37- single, double or triple-positive endosomes was conducted by manually selecting ROIs using ImageJ; line scan analyses and fluorescence intensity profiles were also obtained using ImageJ.

### Statistical Analysis

Data are presented as mean ± SEM. Statistical significance was determined using GraphPad Prism v8 (GraphPad, La Jolla, CA, United States). Normality tests were performed with D’Agostino-Pearson omnibus tests. For non-normally distributed data, Kruskal-Wallis tests were used. Normally distributed data sets were analyzed using unpaired Student’s t test, one-way ANOVA followed by Dunnett post-test, or two-way ANOVA followed by Bonferroni post-test when comparing two groups, more than two groups, or at least two groups under multiple conditions, respectively. Differences were considered significant at *p* < 0.05.

## Results

### Activation of Follicle-Stimulating Hormone Receptor by Thiazolidinone and Benzamide LMW Compounds Increases cAMP Production

Three distinct LMW FSHR ligands were used in this study; two Thiazolidinones, termed here as T1 and T2 (previously Compound 5 (Yanofsky et al., 2006;Sriraman et al., 2014) and Compound 3 (Arey et al., 2008), respectively) and one Benzamide derivative (B3) (Figure 1A). B3 is distinct from previously reported FSHR-selective compounds of this class. It is an enantiomer of Compound 9k (Yu et al., 2014). We first compared the ability of the compounds B3, T1 and T2 to induce increases in cAMP compared to FSH, in our system of HEK 293 cells expressing human FSHR. Gαs/cAMP is the primary G protein signal pathway of FSHR and is regulated at the VEE by APPL1 (Sposini et al., 2017). Two cAMP bioassays were used, one based on a bioluminescence resonance energy transfer (BRET) tagged cAMP effector Epac, and the other directly measuring cAMP levels via HTRF, both of which we have previously employed to study gonadotropin hormone receptor signaling (Ayoub et al., 2015; Sposini et al., 2017). Dose response curves obtained from area under the curves across a kinetic window of 0–20 min using the BRET-based assay demonstrated that both B3 and T1 were significantly less potent than FSH, as indicated by their EC_50_ values, and only T1 exhibited a higher efficacy, expressed as Emax compared to FSH (Figure 1B; Table 1). Based on this observation, for all subsequent experiments doses of compounds that were able to generate maximal responses, namely 10 nM for FSH and 10 μM for B3 and T1 were used.

**TABLE 1:** Potency (pEC50) and efficacy (E_max_) displayed by FSH, B3 and T1 in BRET CAMYEL assays.

Ligand	pEC_50_ ± SEM (pM)	E_max_ ± SEM (%)
FSH	8.3 ± 0.1	101.0 ± 4.4
B3	5.3 ± 0.2****	83.46 ± 8.1*
T1	5.9 ± 0.1	119.2 ± 4.5

p > 0.05, non-significant (ns); p < 0.05, *; p < 0.0001, ****.

**TABLE 2:** Co-localization of FSHR with endosomal markers via TIR-FM.

Ligand	N endosomes	% FSHR-APPL1	% FSHR-EEA1
FSH	0.274 ± 0.019, n = 24	42.47 ± 4.043, n = 12	36.38 ± 5.3, n = 12
B3	0.329 ± 0.017, n = 24	46.51 ± 5.306, n = 12	42.32 ± 6.077, n = 12
T1	0.347 ± 0.016, n = 24	56.67 ± 6.264, n = 10	37.36 ± 4.854, n = 11

We then assessed the levels of cAMP produced following acute stimulation of FSHR by the LMW compounds and FSH, using a HTRF-based assay. As expected, T2 did not induce any cAMP response, as this compound has been previously described as an antagonist of FSHR (Arey et al., 2008). While B3 increased intracellular levels of cAMP to similar levels as the levels induced by FSH, T1-induced cAMP was almost two-fold higher than FSH (Figure 1C) and consistent with the BRET-based assay over distinct time points (Supplementary Figure S1). Taken together, these data demonstrate that both B3 and T1 can activate the Gαs-cAMP pathway via FSHR.

### Profiling of Low Molecular Weight Compounds on Follicle-Stimulating Hormone Receptor Endosomal Trafficking and Post-Endocytic Sorting

**FIGURE 2:**
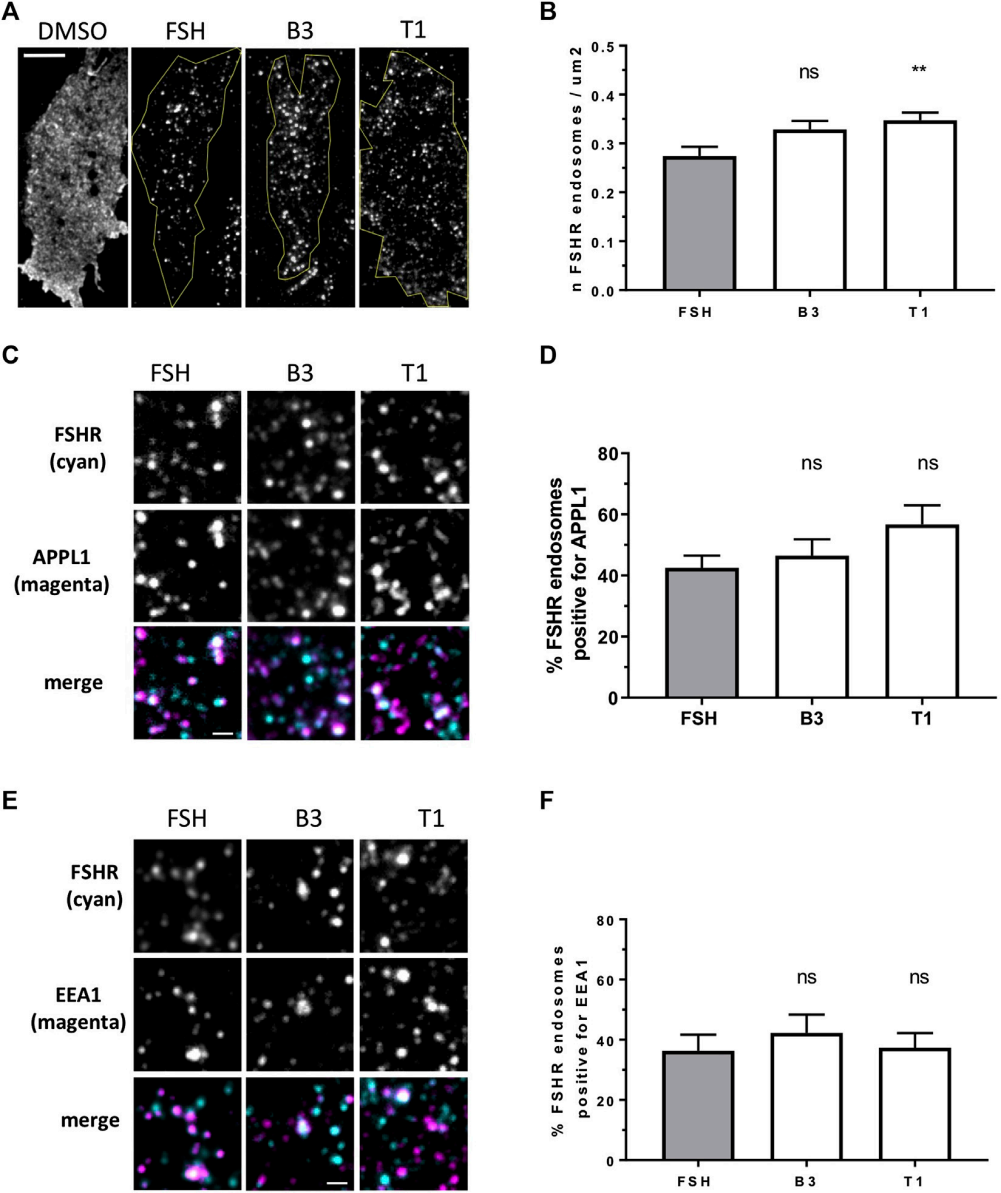
LMW agonists do not alter the endosomal organization of FSHR **(A)** Representative TIR-FM images of FLAG-FSHR expressing HEK 293 cells treated with either DMSO, FSH (10 nM), B3 or T1 (10 μM) for 5 min. Scale bar = 10 μm. **(B)** Quantification of FSHR endosomes in stimulated cells from **(A)**; n = 24 cells/condition collected across three independent experiments. One-way ANOVA: **p < 0.01. **(C)** TIR-FM images of FLAG-FSHR (cyan) and endogenous APPL1 (magenta) in cells stimulated with either FSH (10 nM), B3 or T1 (10 μM) for 5 min. Scale bar = 1 μm. **(D)** Quantification of FSHR endosomes positive for APPL1 from **(C)**; n = 10–12 cells/condition collected across three independent experiments. **(E)** TIR-FM images of FSHR (cyan) and endogenous EEA1 (magenta) in cells stimulated with either FSH (10 nM), B3 or T1 (10 μM) for 5 min. Scale bar = 1 μm. **(F)** Quantification of FSHR endosomes positive for EEA1 from **(E)**; n = 11–12 cells/condition collected across three independent experiments.

Internalization and post-endocytic trafficking of FSHR is an important mechanism to spatially direct its signal activity. We first assessed the ability of the three LMW compounds to induce FSHR endocytosis using live-cell imaging confocal microscopy. Qualitative inspection of cells before and after treatment with DMSO, FSH, B3, T1 or T2 revealed FSHR translocation from the plasma to intracellular vesicular structures upon exposure to FSH, B3 and T1, but not to DMSO or T2 (Supplementary Figure S2). Following ligand-induced internalization, GPCRs such as FSHR are primarily sorted to VEEs over EEs (Jean-Alphonse et al., 2014; Sposini et al., 2017). Furthermore, we have also demonstrated that as the VEE/APPL1 compartment is primarily located in the peripheral region of cells (Sposini et al., 2017), total internal reflection-fluorescence microscopy (TIR-FM) imaging was employed to further assess the organization of internalized FSHR to the VEE and EE following stimulation with either B3 or T1. FSHR expressing cells were stimulated with either FSH, B3 or T1, all of which were able to induce receptor internalization into endosomal compartments. Interestingly, T1-induced FSHR internalization generated a significantly higher number of FSHR endosomes compared to FSH (Figures 2A,B; Table 2).

To examine the endosomal population that each LMW compound was targeting FSHR to, cells were treated with antibodies for APPL1, which is currently the only known protein that localizes to the VEE, yet only localizes to a subpopulation of this compartment (Sposini et al., 2017), and EEA1 a classic marker of the EE. Consistent with our acute cAMP measurements, cells were treated with each compound for 5 min; a time-point we have previously demonstrated that VEE-directed receptors reach steady state in terms of VEE occupancy and endosomal organization (Jean-Alphonse et al., 2014; Caengprasath et al., 2020). TIR-FM imaging and co-localization analysis demonstrated that FSHR endosomes, following stimulation with FSH, co-localized with APPL1 (∼40%) and EEA1 endosomes (∼35%), which is a profile previously observed for other VEE-targeted GPCRs. In contrast, EE-directed GPCRs exhibit >70% co-localization with EEA1 (Jean-Alphonse et al., 2014; Sposini et al., 2017; Caengprasath et al., 2020). B3 and T1 did not significantly differ in the proportion of APPL1- and EEA1-positive FSHR endosomes compared to FSH (Figures 2C–F; Table 2), suggesting that these allosteric compounds were not altering FSHR endosomal targeting.

**FIGURE 3:**
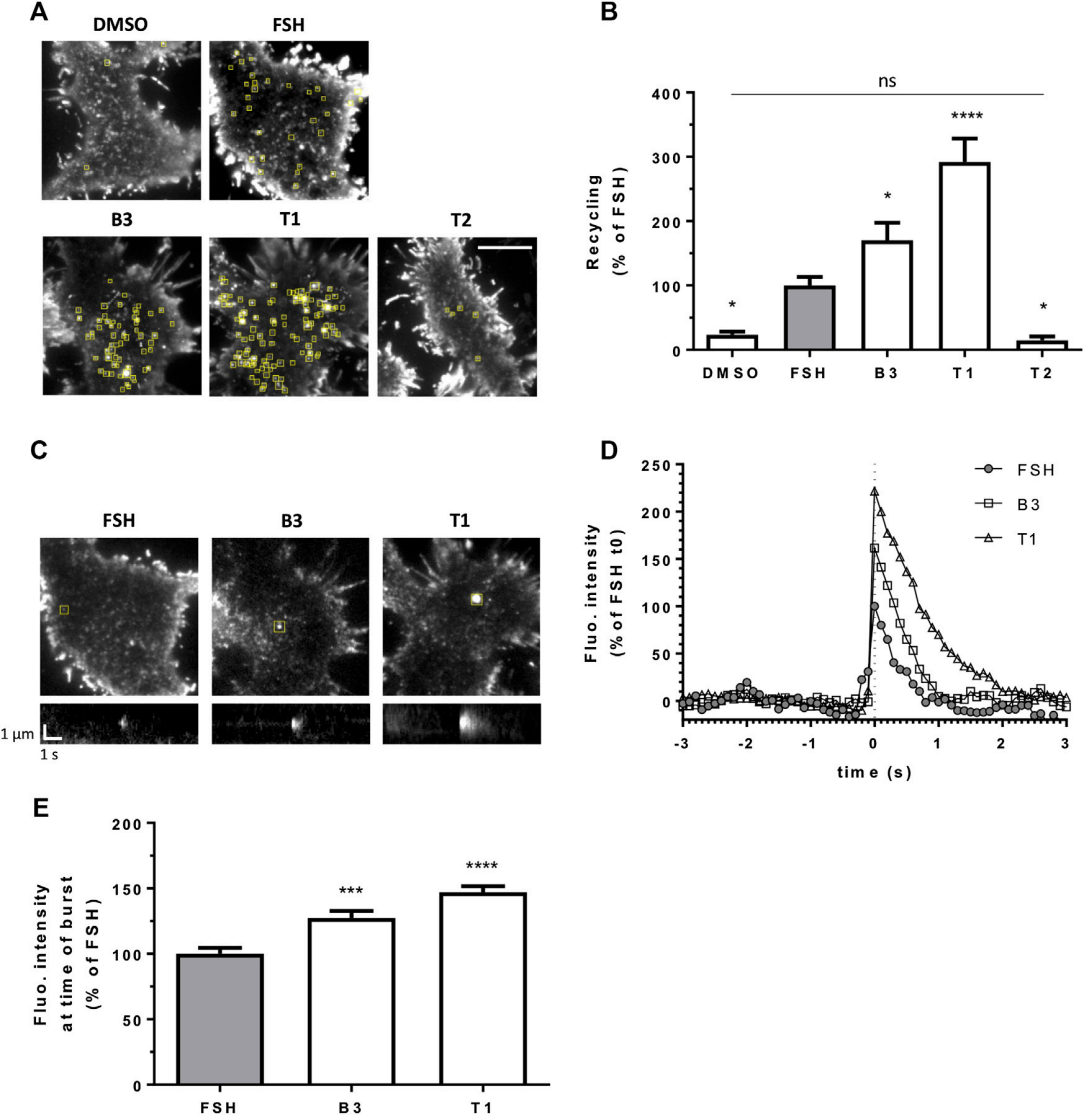
LMW agonists of FSHR induce a greater exocytic rate than FSH. **(A)** Maximum intensity projections from SEP-FSHR expressing HEK 293 cells imaged live by TIR-FM before and after 5-min stimulation with either DMSO, FSH (10 nM), B3, T1 or T2 (10 μM). Yellow squares indicate exocytic events detected in the corresponding TIR-FM movies (Supplementary Movies S1–S5). Scale bar = 10 μm. **(B)** Quantification of the number of FSHR exocytic events from **(A)**; n = 7–11 cells/condition collected across at least three independent experiments. One-way ANOVA: *p < 0.05, ****p < 0.0001. **(C)** Images (top) and kymographs (bottom) of representative FSHR exocytic events (yellow squares) detected after 5-min stimulation with either FSH (10 nM), B3 or T1 (10 μM). **(D)** Fluorescence intensity of the representative FSHR exocytic events shown in **(C)**. Data represent average fluorescence values within an ROI drawn around each event measured 3 s (30 frames) before and after event burst (t0) minus background fluorescence (= fluorescence measured in the ROI during the 30 frames before event burst (t0) was averaged and subtracted from fluorescence values at each frame); values were normalized considering FSH fluorescence at t0 = 100%. **(E)** Fluorescence intensity at time of burst of n = 212, 326, and 328 exocytic events from 11, 9 and 9 cells treated with FSH, B3 and T1, respectively, from **(B)**; values represent maximum intensity fluorescence minus background fluorescence in each ROI at time of event burst; values were normalized considering FSH = 100%; Kruskal-Wallis test: ***p < 0.001, ****p < 0.0001.

After its internalization into endosomes, FSHR is targeted to a rapid recycling pathway back to the plasma membrane. We have previously described an approach to visually quantify LHR recycling events in live cells and in real time, using TIR-FM (Sposini et al., 2017). Here we used the same approach by N-terminally tagging FSHR with the pH sensitive GFP variant Super Ecliptic Phluorin (SEP). Quantification of exocytic events revealed that in agonist-stimulated cells the number of events was significantly increased compared to vehicle (DMSO) treated cells, and that both B3 and T1 induced an increase in the number of exocytic events compared to FSH, namely 1.5 fold increase by B3 and remarkably, almost a three fold increase for T1 (Figures 3A,B; Supplementary Movies S1–S4). Conversely, the number of FSHR recycling events following treatment with the antagonist T2 was not significantly different to basal and significantly lower than the events induced by FSH (Figure 3A,B; Supplementary Movie S5).

We also observed that individual exocytic events induced by B3 and T1 were generally bigger and brighter than those generated by stimulation with FSH. This qualitative observation was confirmed by measuring the fluorescence intensity of recycling events at the time of burst, or opening of the vesicle at the plasma membrane, which was indeed significantly higher for both B3 and T1, compared to FSH (Figures 3C–E). These studies suggest that B3 and T1 affect the recycling properties of FSHR, increasing its recycling rates, particularly for T1, and the size of recycling vesicles compared to FSH.

### Low Molecular Weight Agonists of Follicle-Stimulating Hormone Receptor Activate Endosomal Gαs/cAMP Signaling

**FIGURE 4:**
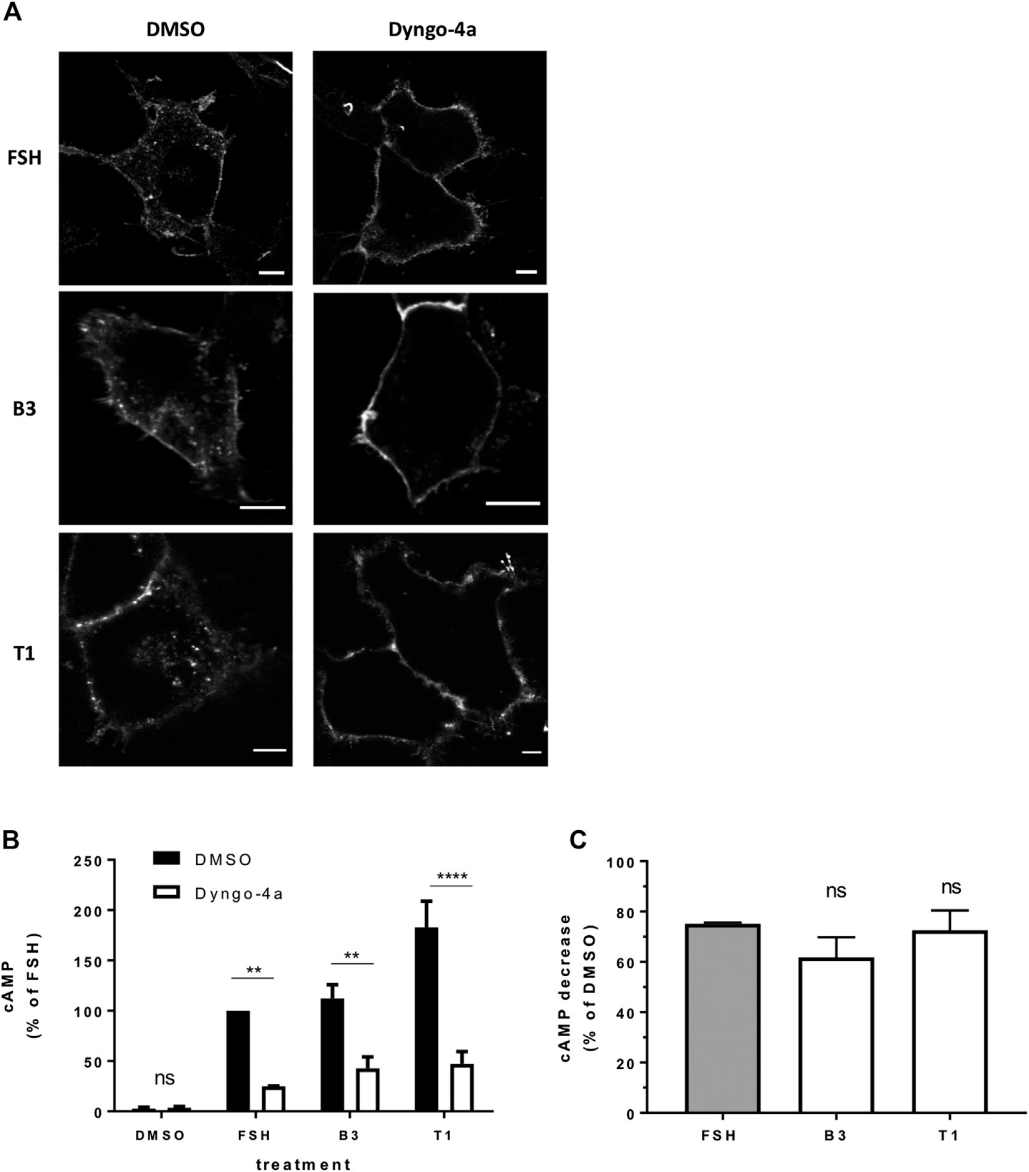
FSHR requires receptor internalization to induce cAMP signaling in response to either FSH or LMW agonists. **(A)** Confocal images of FLAG-FSHR stably expressing HEK 293 cells pre-treated with either DMSO or Dyngo-4a (30 μM, 30 min) and stimulated with FSH (10 nM), B3 (10 μM) or T1 (10 μM). Scale bar = 5 μm. **(B)** Intracellular levels of cAMP measured in cells via HTRF expressing FLAG-FSHR pre-treated with either DMSO or Dyngo-4a (30 μM, 30 min) and stimulation either DMSO, FSH (10 nM, 5 min), B3 or T1 (10 μM, 5 min). Data are expressed as cAMP levels normalized to FSH treatment (DMSO). Two-way ANOVA: **p < 0.01, ****p < 0.0001. **(C)** Percentage decrease in cAMP levels following Dyngo-4a pre-treatment calculated from **(B)**. n = 3 independent experiments.

We have previously demonstrated that FSH-induced signaling activity is regulated by VEE machinery (Jean-Alphonse et al., 2014; Sposini et al., 2017). FSHR internalization was inhibited with pre-treatment of cells with Dyngo-4a, which inhibits the GTPase dynamin (McCluskey et al., 2013). Inhibition was confirmed via confocal imaging of FSHR following stimulation with FSH, B3 or T1 (Figure 4A). Dyngo-4a significantly inhibited FSH-induced cAMP signaling when measured via HTRF-based assay (Figure 4B; Supplementary Figure S3; Supplementary Tables S1, S2). Furthermore, both B3 and T1-induced cAMP signaling was equally blunted following Dyngo-4a treatment, indicating that FSHR internalization was required to achieve a full cAMP response from both the natural and synthetic ligands (Figure 4C).

**FIGURE 5:**
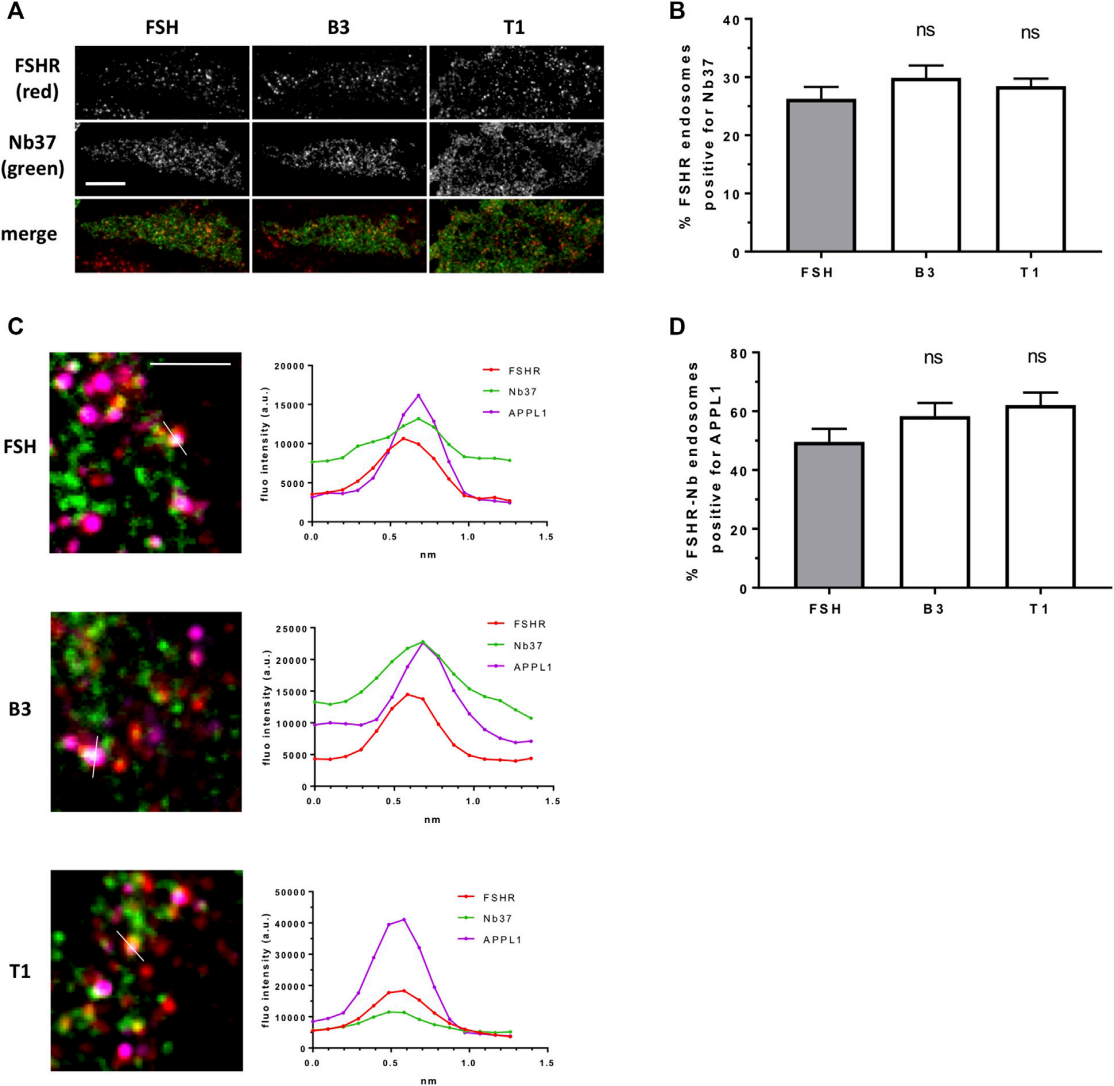
LMW compounds induce Gαs activation from FSHR endosomes. **(A)** TIR-FM images of FLAG-FSHR (red) expressing cells transfected with Nb37-GFP (green) treated with either FSH (10 nM), B3 or T1 (10 μM) for 5 min. Scale bar = 10 μm. **(B)** Quantification of FSHR endosomes from **(A)**; n = 24 cells/condition in three independent experiments. **(C)** TIR-FM images (top) of FLAG-FSHR (red), Nb37-GFP (green) and endogenous APPL1 (magenta) in cells stimulated with either FSH, B3 or T1 for 5 min. Scale bar = 3 μm. Line scan analysis of fluorescence intensity (bottom) is shown for one endosome per condition showing FSHR-Nb37-APPL1 colocalization (representative of 233, 302, 316 endosomes for FSH, B1, T3, respectively). **(D)** Quantification of FSHR-Nb37 endosomes positive for APPL1 after 5-min stimulation with either FSH, B3 or T1; n = 11–12 cells/condition from **(C)** from three independent experiments.

Our data above suggests T1 induces a more efficacious FSHR-mediated cAMP response, a greater number of endosomes and an increased exocytosis rate, yet, all compounds were equally sensitive to inhibition of receptor internalization. Therefore, to determine if the increased cAMP signaling by T1 was due to a greater proportion of active Gαs signaling from endosomes we employed the GFP-tagged nanobody37 (Nb37-GFP) which recognizes the active, nucleotide free conformation of Gαs, and was previously employed by us and others to directly visualize G protein signaling from endosomes (Irannejad et al., 2013; Sposini et al., 2017). TIR-FM imaging revealed that a subpopulation of FSHR endosomes, following FSH stimulation, were positive for Nb37, indicating the presence of active Gαs at endosomal compartments. Moreover, stimulation of cells with B3 or T1, did not alter the proportion of Gαs signaling endosomes compared to FSH (Figures 5A,B). We have previously demonstrated that a population of LHR signaling endosomes are positive for APPL1 (Sposini et al., 2017). Likewise, approximately half of the active signaling endosomes induced by FSH were positive for APPL1. This proportion was not significantly different in cells stimulated with either B3 or T1 compared to FSH (Figures 5C,D; Table 3).

**TABLE 3:** Co-localization of FSHR endosomes with Nb37 and APPL1 via TIR-FM. Mean ± SE.

Ligand	% FSHR-Nb37	% FSHR-Nb37-APPL1
FSH	26.28 ± 2.029, n = 23	46.37 ± 4.552, n = 12
B3	29.89 ± 2.119, n = 24	49.96 ± 6.117, n = 12
T1	28.43 ± 1.307, n = 24	50.13 ± 5.504, n = 10

### Effect of Low Molecular Weight Compounds on APPL1-Regulated Follicle-Stimulating Hormone Receptor Signaling and Recycling

We have previously demonstrated that APPL1 plays two key and distinct functions in regulating FSHR activity: it is required for receptor recycling and negative regulation of endosomal cAMP when stimulated with its natural ligand (Sposini et al., 2017). One possibility for the enhanced signaling and recycling induced by the LMW ligands, and T1 in particular, is altered regulation by APPL1.

**FIGURE 6:**
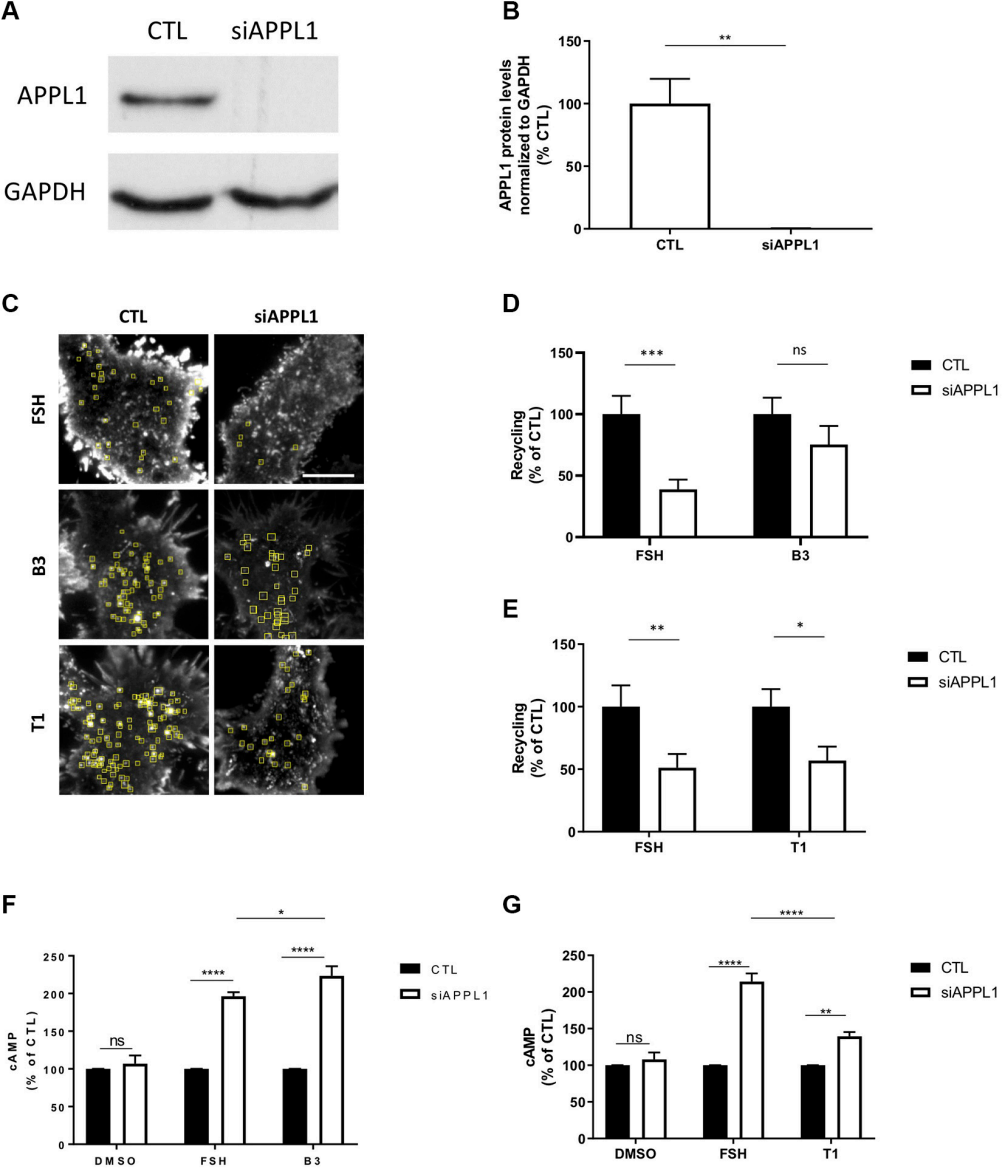
LMW compounds differentially affect APPL1-regulated FSHR recycling and cAMP signaling. **(A)** Western blot showing total cellular levels of APPL1 from cells treated with scramble (CTL) or APPL1 siRNA (siAPPL1). GAPDH was used as a loading control. **(B)** Quantification of APPL1 protein levels normalized to GAPDH protein levels from **(A)**, and expressed as % of scramble, n = 3 independent experiments, *t*-test: **p < 0.01. **(C)** Maximum intensity projections from SEP-FSHR expressing cells imaged live by TIR-FM following transfection with either scramble (CTL) or APPL1 siRNA (siAPPL1) and stimulated with either FSH (10 nM), B3 or T1 (10 μM) for 5–20 min. Scale bar = 5 μm. **(D–E)** Quantification of the number of FSHR recycling events. n = 21–24 cells per condition collected across four independent experiments for **(D)** and n = 13–18 cells per condition collected across three independent experiments for **(E)**. Two-way ANOVA: *p > 0.05, **p < 0.01, ***p < 0.001. **(F,G)** Intracellular levels of cAMP measured in cells stably expressing FLAG-FSHR following transfection with either scramble (CTL) or APPL1 siRNA (siAPPL1). Cells were stimulated with either DMSO, FSH (10 nM) or B3 (10 μM) **(F)** or DMSO, FSH (10 nM) or T1 (10 μM) **(G)** for 5 min. n = 5 **(F)** or 3 **(G)** independent experiments. Two-way ANOVA: *p < 0.05, **p < 0.001 ****p < 0.0001.

To test this hypothesis, we depleted APPL1 by siRNA-mediated knock-down in FSHR expressing cells (Figures 6A,B) and measured ligand-induced recycling and cAMP generation by FSHR. APPL1 depletion significantly inhibited FSH-dependent recycling of FSHR, and resulted in a ∼2 fold increase in FSH-induced cAMP levels, as previously reported (Figures 6C–G) (Sposini et al., 2017). Unexpectedly, APPL1 depletion did not significantly inhibit FSHR recycling in cells stimulated by B3, while T1-induced recycling was inhibited to a similar level as FSH (Figures 6C–E; Supplementary Movies S6–S8). Although loss of APPL1 did not impact B3-induced exocytosis of FSHR, this was not due to *de novo* receptor biogenesis, as pre-treatment with cycloheximide had no effect on plasma membrane insertion events of FSHR when stimulated with either B3- or FSH (Supplementary Figure S4). In an opposing manner to exocytic profiles, B3-induced cAMP was increased following APPL1 knock-down to a similar level as FSH (Figure 6F), while T1-induced cAMP signaling was only marginally increased and significantly lower than that of FSH (Figure 6G). Overall, this suggests that APPL1 has a reduced ability to regulate B3-induced recycling while negative regulation of endosomal signaling is maintained. In contrast, T1-induced recycling is APPL1-regulated, while its endosomal signaling exhibits a reduced sensitivity to APPL1 knockdown.

## Discussion

The spatial control of GPCR signaling has emerged as a mechanism for generating both signal diversity and for cells to decode complex signaling profiles. While it is well understood that GPCR internalization and post-endocytic sorting to distinct cellular fates is a highly regulated and multi-step system (Hanyaloglu and von Zastrow, 2008; Bowman and Puthenveedu, 2015), a similar level of exquisite control in driving and regulating endosomal signaling is also emerging. Here, by using previously reported and novel LMW ligands of the FSHR, we provide evidence that the distinct FSHR endosomal signaling and trafficking functions of a key protein of the VEE, APPL1, can be uncoupled in a divergent manner.

In this study, we employed two chemically distinct FSHR-selective ligands, a thiazolidinone (T1) and a benzamide compound (B3); chemical classes that have been reported to be allosteric activators of FSHR (Arey et al., 2008; Sriraman et al., 2014; Yu et al., 2014). T1 has been previously characterized, including an ability to induce preovulatory follicular development in immature rats, however, it displayed poor pharmacokinetic parameters *in vivo* (Sriraman et al., 2014). The benzamide class of FSHR ligands exhibit improved pharmacokinetics, yet their ability to induce follicle development has not been fully characterized (Yu et al., 2014). The current study employed a benzamide analog that has not been previously reported, however, the lower potency of B3 and T1 compared to FSH that we observed, are consistent with previous reports for these classes of LMW agonists. The significantly enhanced Emax of T1 suggests FSHR can be activated beyond the native ligand FSH. Similar increases in maximal response of β-arrestin recruitment to FSHR by T1 was previously reported (Jiang et al., 2014), and is also consistent with our observations that T1 can enhance the number of FSHR endosomes compared to FSH. In the current study, enhanced cAMP is observed when cAMP signaling was directly measured at acute time points (5 min) and via the BRET based CAMYEL sensor over a 20 min period. However, there was no difference in Emax observed between FSH and T1 following a 1-h stimulation, when cAMP was also measured by HTRF in rat granulosa cells (Sriraman et al., 2014), although one cannot also exclude differences in regulation between human and rodent FSHR. As we have previously demonstrated that acute signaling of gonadotropin hormone receptors are tightly regulated by receptor endocytosis, we focused on this kinetic window for subsequent experiments to assess endosomal signaling and trafficking properties of these ligands.

While T1 exhibited enhanced efficacy in cAMP signaling of FSHR, both B3 and T1 could induce a greater rate of exocytosis, with an increased brightness and size for each individual fusion event, or “puff”. The latter suggests that a greater number of receptors are recycled within each vesicle. Again, this was more pronounced for T1, exhibiting nearly a three-fold increase in exocytosis rate. While the increased number of T1-induced FSHR endosomes may in part explain the enhanced recycling, it is also possible that the increased maximal cAMP responses induced by this compound play a role, in view of our findings that LHR recycling is driven by its cAMP signaling (Sposini et al., 2017). However, the enhanced recycling of B3 does not correlate with its cAMP signaling, compared to FSH, nor its endocytic trafficking. Indeed, the endosomal organization of FSHR was not significantly different between any of the agonists (synthetic and hormone), indicating that the initial molecular steps that target FSHR toward VEEs may be unaltered. For LHR, this targeting step is mediated by the PDZ protein GIPC (GAIP-interacting protein C terminus) via interactions with its C-terminal tail early during clathrin-mediated endocytosis (Jean-Alphonse et al., 2014). The loss, or disruption, of this association between receptor and GIPC directs LHR to physically larger EEs and whereby the majority of receptor endosomes (∼70%) co-localize with EE markers (Jean-Alphonse et al., 2014). While GIPC is required for FSHR-mediated MAP kinase signaling (Jean-Alphonse et al., 2014), the role of GIPC in VEE targeting of FSHR needs to be further characterized given its C-terminal tail lacks a PDZ ligand. APPL1 is known to associate with GIPC (Lin et al., 2006), and APPL1 can also directly associate with FSHR (Nechamen et al., 2004). This association may be a clue for understanding the surprisingly divergent sensitivities these compounds induce in FSHR-mediated regulation by this VEE adaptor protein.

As described above, APPL1 plays two key functions in the VEE; driving plasma membrane recycling and negative regulation of G protein signaling (Sposini et al., 2017; Caengprasath et al., 2020). These two roles are further highlighted as distinct functional properties of APPL1 through our findings in this study with FSHR LMW ligands, which exhibited differential sensitivity to these two properties in an opposing manner. All agonists, including FSH, exhibited a similar requirement for receptor internalization for its acute cAMP signaling, and further supported by the direct visualization of active Gαs signaling endosomes following ligand-treatment. As the proportion of endosomal signaling was similar across ligands, we proposed that the enhanced cAMP signaling by T1 may be due to altered regulation by APPL1. Indeed, when APPL1 cellular levels were depleted, the cAMP signaling induced by this compound was only marginally increased compared to the more than two-fold increase in cAMP signaling induced by FSH. This suggests that the enhanced efficacy of T1 is due to a reduced ability of APPL1 to “switch off” its endosomal signaling. In contrast, the recycling of FSHR by T1 was APPL1-dependent, suggesting the APPL1 mechanism that mediates this function is maintained, and potentially that the enhanced cAMP signaling from FSHR underlies the augmented receptor exocytosis by T1. While we know that receptor-mediated cAMP/PKA signaling leads to phosphorylation of APPL1 on serine 410 to drive LHR recycling from the VEE (Sposini et al., 2017), the mechanisms underlying its negative regulation is unknown, and a current area of investigation, although we have demonstrated that it does not require APPL1 phosphorylation by PKA and thus distinct from the process mediating recycling (Sposini et al., 2017). In contrast to T1, B3-driven cAMP signaling was negatively regulated by APPL1, potentially explaining why the efficacy of this compound is similar to FSH. Unexpectedly, B3-mediated FSHR recycling was independent of APPL1. Combined with the ability of this compound to enhance receptor recycling over FSH, suggests this ligand may enable the receptor to sort to distinct recycling pathways, which would need to be defined in future studies. Although the endosomal organization of internalized FSHR following B3-mediated activation was similar to FSH, knockdown of APPL1 perhaps reroutes the B3-activated FSHR to alternate recycling pathways, however, the endosomal organization of FSHR was similar between FSH, B3 and T1 and we have demonstrated that loss of APPL1 does not alter the endosomal organization of LHR like knockdown of GIPC (Sposini et al., 2017). Another possibility is that APPL1-independent sorting could still occur from the VEE. A precedence for this, is the ability to alter β2-adrenergic receptor recycling from a regulated/PDZ-dependent to a default/PDZ-independent pathway by via distinct sites on its C-tail (Hanyaloglu and von Zastrow, 2007; Vistein and Puthenveedu, 2013). This “switch” from sorting receptors from a regulated to a default recycling pathway both occur from the EE, and there is evidence that these two forms of recycling can occur from the same endosome yet emanate from distinct recycling tubules (Puthenveedu et al., 2010). VEEs are ∼400 nm (Jean-Alphonse et al., 2014), thus imaging recycling tubules in live cells has been challenging due to resolution limitations, although we have reported that LHR signaling from VEEs can occur from microdomains within a single endosome via super-resolution imaging in fixed cells (Sposini et al., 2017). Thus, B3 could direct sorting of FSHR into a default recycling pathway from VEE/APPL1 endosomes. Overall, these compounds have demonstrated that these APPL1 functions regulate the receptor via highly distinct mechanisms. This is intriguing given APPL1 has been shown to interact with the first intracellular loop of FSHR (Nechamen et al., 2004) and could suggest distinct interaction sites and/or conformational complexes of FSHR and APPL1 determine these functions. This could be akin to how arrestin mediates multiple functions of GPCRs through associations between the GPCR core and C-tail (Kumari et al., 2016; Thomsen et al., 2016).

These ligands have provided novel tools to further probe these regulatory features of the VEE and potentially toward understanding its downstream impact. At present how the VEE impacts FSHR, or LHR, signaling in the ovary or testes is unknown. While T1 could induce follicular development in rats and estradiol production in primary human granulosa cells, interestingly with a greater Emax than FSH (Sriraman et al., 2014), it is important for future studies to apply these ligands to understand their impact on spatially-directed signaling in physiological human systems, including the ability of these allosteric molecules to modify FSH-induced responses. In addition, binding kinetic profiles of these small molecules may also shed light on the distinct endosomal signaling and trafficking properties of these ligands, although factors such as ligand potency and residency time can also poorly correlate with sustained endosomal signaling profiles of GPCRs where the environment of the lumen could facilitate rebinding of the ligand (Sposini and Hanyaloglu, 2017). At least for LHR, the trafficking of rodent and human receptors is very different (Nakamura et al., 2000; Hirakawa et al., 2003) and so far a clinical study of an FSHR LMW agonist failed to induce follicular development in healthy volunteers, although only low doses were assessed (Gerrits et al., 2016). Given that the majority of the FSHR-induced acute cAMP signaling is endosomal, it is likely these early events would have key physiological functions. Indeed, we have recently demonstrated that it is the acute endosomal signaling of the free fatty acid receptor 2 (a GPCR that traffics to the VEE), which mediates its ability to secrete anorectic gut hormone (Caengprasath et al., 2020). The ability to pharmacologically target endosomal signaling has been demonstrated as a potential therapeutic strategy in pain management (Thomsen et al., 2018). However, one could also envisage that the distinct properties of these LMW ligands, such as bypassing APPL1-dependent negative regulation of signaling, may be advantageous in certain clinical conditions such as patients who are poor FSH responders when undergoing ART.

Compartment or location bias in signaling, not only refers to signaling between plasma membrane and endomembrane, but spatial control across distinct intracellular compartments, including between distinct endosomal compartments (Irannejad et al., 2017; Sposini et al., 2017; Kunselman et al., 2020). Our study potentially demonstrates an additional level of location bias within a single compartment, via the ability of these LMW ligands to differentially engage the VEE machinery APPL1 for distinct functions. Whether additional, as yet unknown, VEE proteins could also be altered, remains to be determined. As we uncover both the detailed mechanisms and downstream functions of this compartment to FSHR activity, it is possible that future FSHR ligand screens that leverage endosomal signaling capacity and APPL1-sensitivity could “customize” the FSHR activity for specific applications.

## Data Availability Statement

The raw data supporting the conclusions of this article will be made available by the authors, without undue reservation.

## Author Contributions

SS, FDP, RR, and NS performed experiments under supervision of AH, ER, and DP. HY, SP, and SN provided the FSHR LMW compounds. AH conceived the study and with SS, FDP, and ER designed research. SS, FDP, and RR analyzed data. SS, FD, and AH wrote the paper. All authors read and approved final manuscript.

## Funding

This work was supported by a Biotechnology and Biological Sciences Research Council (BBSRC) project grant awarded to AH (BB/S001565/1) and a Society for Endocrinology Early Career Grant to SS. The Facility for Imaging by Light Microscopy (FILM) at Imperial College London is part-supported by funding from BBSRC grant BB/L015129/1. ER is supported by the French National Research Agency under the program “Investissements d'avenir” Grant Agreement LabEx MabImprove: ANR-10-LABX-53; “ARD2020 Biomédicaments” and “APR-IR MODUPHAC” grants from Région Centre Val de Loire. FDP was a recipient of a doctoral fellowship from INRA and Région Centre.

## Conflict of Interest

HY, SP, and SN were employees of TocopheRx when reagents were shared. Author SN is employed by the company Mitobridge Inc. Author HY is employed by the company Canwell Pharma.

The remaining authors declare that the research was conducted in the absence of any commercial or financial relationships that could be construed as a potential conflict of interest.
