# Supplementary material for: Pharmacological Programming of Endosomal Signaling Activated by Small Molecule Ligands of the Follicle Stimulating Hormone Receptor
Source: Front Pharmacol. 2020 Nov 30;11:593492. doi: 10.3389/fphar.2020.593492 (PMC7734412; doi:10.3389/fphar.2020.593492)
Supplement: Supplementary file 1 [file datasheet1.pdf]

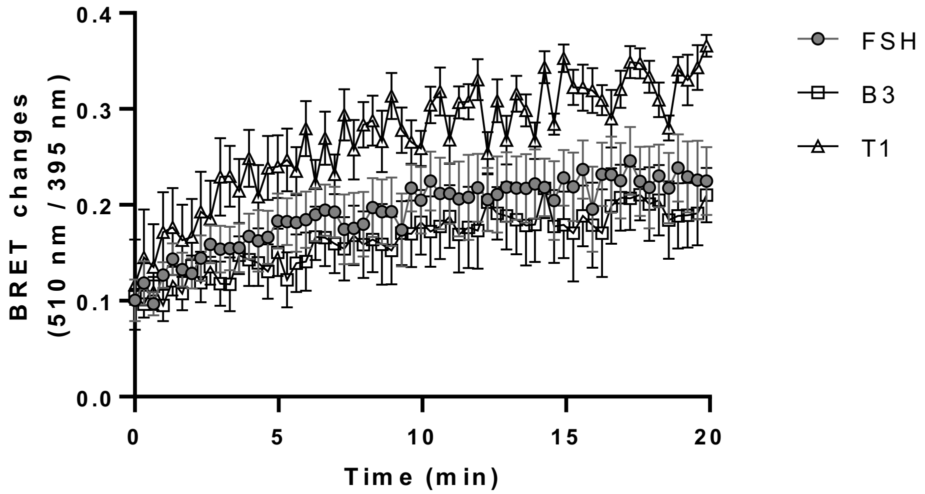

Supplemental Figure 1. Kinetic profiles of cAMP production induced by FSH, B3 and T1. Cells transiently transfected with FSHR and cAMP BRET sensor CAMYEL were stimulated with FSH (3.33 nM), B3 (10 µM) or T1 (10 µM). Signal was immediately recorded for 20 min upon addition of each ligand. The signal obtained from unstimulated cells was subtracted from the signal recorded at each time-point shown in the curve. n= 6 independent experiments.

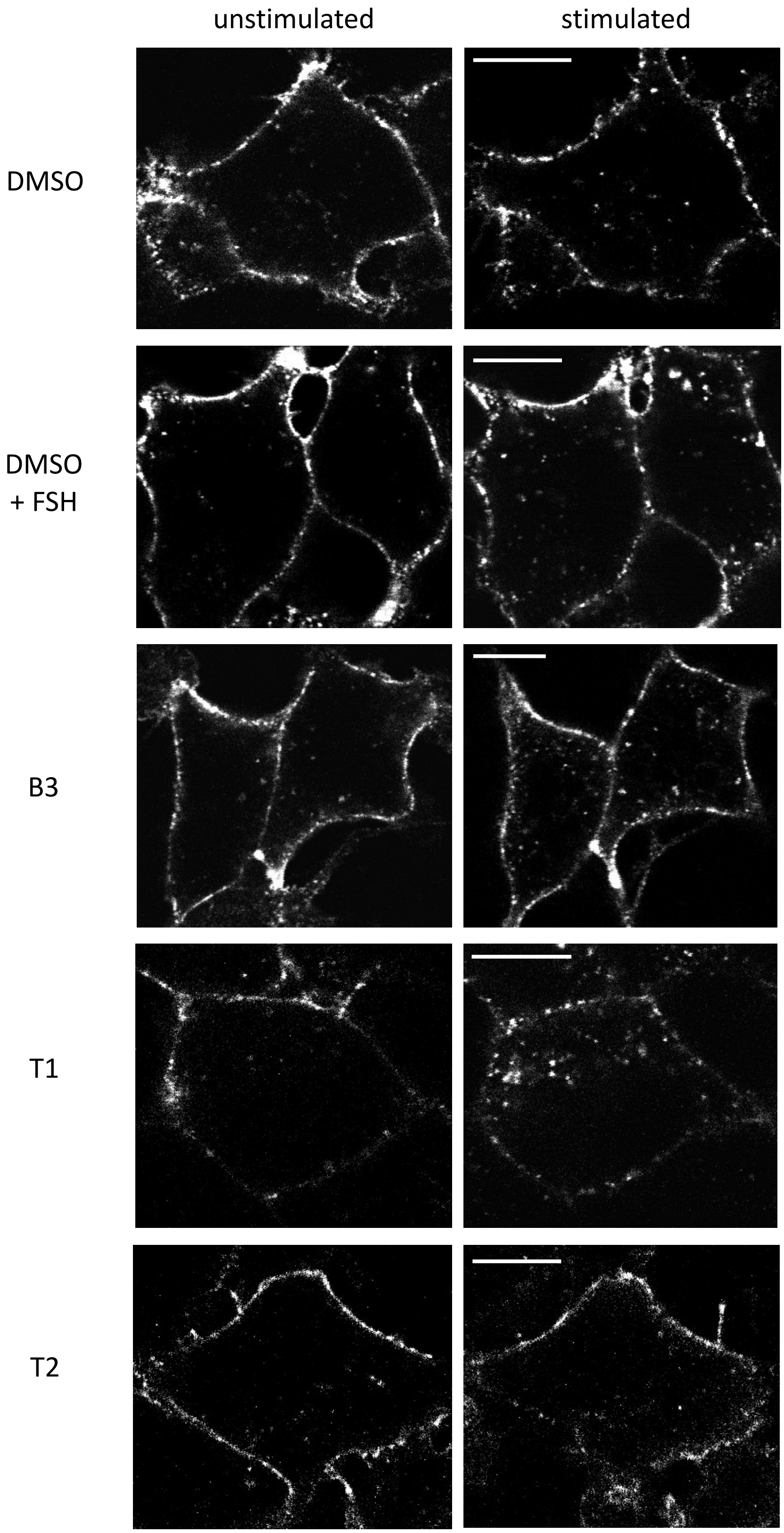

Supplemental Figure 2. FSHR internalization is induced by FSH, B3 or T1 but not DMSO or T2. (A) Representative live confocal images of FLAG-FSHR expressing HEK 293 cells before (unstimulated) and after (stimulated) treatment with either DMSO, DMSO + FSH (10 nM), B3 (10 μM) or T1 (10 μM) for 5 min. Scale bar= 10 μm.

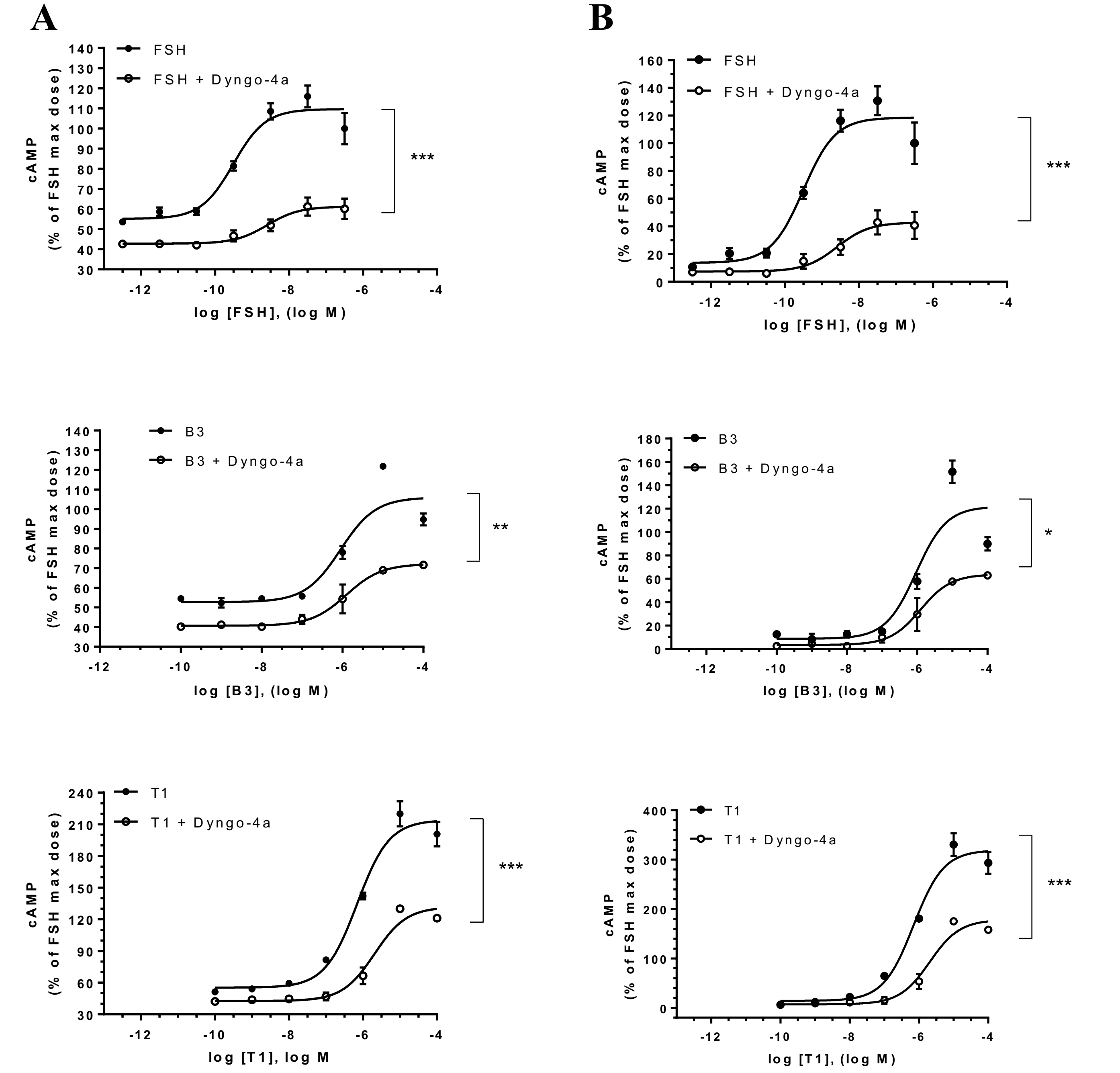

Supplemental Figure 3. FSHR requires receptor internalization to induce cAMP signaling in response to either FSH or LMW agonists. (A-B) Concentration/activity curves for cAMP production induced by FSH, B3 and T1 in the presence/absence of Dyngo-4A (30 µM, 30 min pre-treatment). FSHR-transfected cells were stimulated for 5 min and ligand-induced cAMP production was assayed via HTRF. In B, the signal obtained from unstimulated cells was subtracted from the signal recorded at each dose of ligand shown in the curve. Concentrations of FSH were -12.5 to -6.5 log M while B3 and T1 were -10 to -4 log M. Data are normalized considering the maximal dose of FSH used and in absence of Dyngo-4A as 100%. n = 3 independent experiments. T test: **p< 0.01, ***p<0.001.

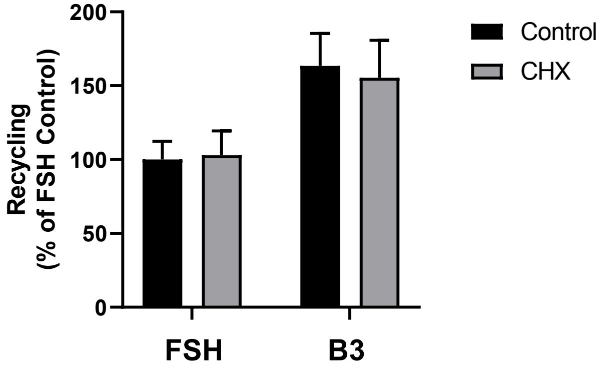

Supplemental Figure 4. FSHR exocytic events induced by FSH or B3 are not affected by cycloheximide. Number of plasma membrane insertion events measured by TIR-FM in HEK 293 cells stably expressing SEP-FSHR, pre-treated with DMSO (control) or cycloheximide (CHX, 10 mg/mL, 90 min) prior to FSH (10 nM) or B3 (10 μM) stimulation. n = 14-15 cells per condition, collected across 2 independent experiments.
