# Supplementary material for: Pharmacological Programming of Endosomal Signaling Activated by Small Molecule Ligands of the Follicle Stimulating Hormone Receptor
Source: Front Pharmacol. 2020 Nov 30;11:593492. doi: 10.3389/fphar.2020.593492 (PMC7734412; doi:10.3389/fphar.2020.593492)
Supplement: Supplementary file 2 [file table1.docx]

Supplemental table 1 – Potency (EC50) and efficacy (E_max_) displayed by FSHR ligands in cells treated with or without Dyngo-4a in HTRF assays.

| **Ligand** | **pEC_50_ + SEM (pM)** | **E_max_ + SEM (%)** |
| --- | --- | --- |
| FSH | 9.3 ± 0.1 | 109.6 ± 2.9 |
| FSH + Dyngo-4a | 8.4 ± 0.3 | 61.1 ± 2.1  *** |
| B3 | 6.0 ± 0.2 | 106.0 ± 4.3  ** |
| B3 + Dyngo-4a | 5.9 ± 0.2 | 72.0 ± 2.7 |
| T1 | 6.2 ± 0.1 | 213.7 ± 6.0 |
| T1 + Dyngo-4a | 5.7 ± 0.1  * | 131.6 ± 5.2  *** |
