# Supplementary material for: Pharmacological Programming of Endosomal Signaling Activated by Small Molecule Ligands of the Follicle Stimulating Hormone Receptor
Source: Front Pharmacol. 2020 Nov 30;11:593492. doi: 10.3389/fphar.2020.593492 (PMC7734412; doi:10.3389/fphar.2020.593492)
Supplement: Supplementary file 3 [file table2.docx]

Supplemental table 2 – Potency (pEC50) and efficacy (E_max_) displayed by FSHR ligands in cells treated with or without Dyngo-4a in HTRF assays, after subtraction of basal values.

| **Ligand** | **pEC_50_ + SEM (pM)** | **E_max_ + SEM (%)** |
| --- | --- | --- |
| FSH | 9.5 ± 0.2 | 118.5 ± 5.9 |
| FSH + Dyngo-4a | 8.6 ± 0.3 | 42.8 ± 4.3  *** |
| B3 | 6.0 ± 0.2 | 121.8 ± 10.8  * |
| B3 + Dyngo-4a | 5.9 ± 0.2 | 63.9 ± 5.6 |
| T1 | 6.2 ± 0.1 | 319.2 ± 12.0 |
| T1 + Dyngo-4a | 5.7 ± 0.1  * | 178.4 ± 10.5  *** |
